# Pharmacokinetic of two oral doses of a 1:20 THC:CBD *cannabis* herbal extract in cats

**DOI:** 10.3389/fvets.2024.1352495

**Published:** 2024-02-23

**Authors:** Chloe Lyons, Katelyn McEwan, Meara Munn-Patterson, Stephanie Vuong, Jane Alcorn, Alan Chicoine

**Affiliations:** ^1^Department of Veterinary Biomedical Sciences, Western College of Veterinary Medicine, University of Saskatchewan, Saskatoon, SK, Canada; ^2^College of Pharmacy and Nutrition, University of Saskatchewan, Saskatoon, SK, Canada

**Keywords:** cannabinoids, CBD -cannabidiol, THC -tetrahydrocannabinol, pharmacokinetics, relative bioavailability, feline

## Abstract

**Objective:**

To determine the pharmacokinetics (PK) of two oral doses of a *Cannabis* herbal extract (CHE) containing 1:20 THC:CBD in 12 healthy Domestic Shorthair cats.

**Methods:**

Single-dose PK were assessed after oral administration of CHE at low or high dose (2 mg CBD + 0.1 mg THC, or 5 mg CBD + 0.25 mg THC per kg bw, respectively; *n* = 6 per group) in fasting cats. Blood samples were drawn up to 48 h following CHE administration. Plasma samples were analyzed for CBD, THC, and metabolites 6-OH-CBD, 7-OH-CBD, 11-OH-THC, and THC-COOH using a previously validated LC–MS/MS method.

**Results:**

CBD and THC were quickly absorbed (mean *T*_max_ of 2.4–2.9 h). Maximum plasma concentrations (*C*_max_) ranged from 36–511 ng/mL and 6.8–61 ng/mL for CBD and THC, respectively. Elimination was initially rapid for both CBD and THC, though a prolonged elimination phase was noted for CBD in some cats (T_1/2 λ_ up to 26 h). Dose-adjusted *C*_max_ and AUC_0-last_ values were not statistically significantly different (*p* > 0.05) between dose groups indicating CBD and THC concentrations increased in a manner proportional (linear) to the dose. Dose-adjusted THC *C*_max_ and AUC_0-last_ were significantly higher than the corresponding dose-adjusted CBD parameters (*p* < 0.01). Low concentrations of the metabolite 6-OH-CBD were quantified but metabolites 7-OH-CBD, 11-OH-THC, and THC-COOH were not detected in any plasma samples. Inter-individual variance was notable. Salivation shortly after dosing was observed in two cats in the high dose group; these animals had substantially lower cannabinoid concentrations than other cats in this group. No adverse clinical signs (including vomiting, change in mentation or other neurological signs) were noted.

**Clinical significance:**

Although cats did not display adverse effects after administration of a single oral dose of 1:20 THC:CBD CHE formulation at 2 or 5 mg CBD/kg bw, observed plasma concentrations were highly variable but generally lower than in dogs receiving the same dose and formulation. Administration of CHE in the fasting state may not optimize CBD absorption, and oral dosing may be challenging when administering an oil-based CHE in some cats.

## Introduction

Phytocannabinoids are compounds derived from plants, *Cannabis sativa* and *Cannabis indica.* Although used in traditional medicine for centuries, medical use of cannabinoids has received increased medical interest with discovery of the endocannabinoid system (1) and its recent legalization in many countries. Cannabinoids bind allosterically to cannabinoid receptors (CB1 and CB2) that are widely distributed throughout the mammalian body (1), making them an attractive therapy for a variety diseases and conditions in both human and veterinary medicine. However, the mechanism of action is not fully understood, and the extent of its therapeutic properties is currently under investigation. There are over 120 cannabinoids, the most common being delta-9-tetrahydrocannabinol (Δ9-THC) and cannabidiol (CBD). THC is widely recognized for its psychotropic effects and thus its toxicity has been extensively studied in both humans and animals; but the drug has also demonstrated to be effective at decreasing symptoms of nausea and vomiting in human patients receiving chemotherapy (2). CBD has been gaining attention as a nutraceutical due to its ability to provide therapeutic benefits without impairing cognition. It has been recognized to effectively reduce seizure severity and frequency in children and young adults (3, 4) and appears promising for similar use in anticonvulsant-resistant epileptic animals (5). Furthermore, CBD has been demonstrated to have anti-inflammatory and immunomodulating properties in models using canine and equine whole blood (6, 7).

When considering potential benefits of their use in veterinary medicine, understanding the pharmacokinetics of cannabinoids in animals is crucial for developing rational dosing regimens. Oral bioavailability of CBD appears to be low in cats, possibly due to its lipophilic structure and potential for first-pass metabolism (8). Differences in pharmacokinetic parameters have been observed both within and between species (9). Felines appeared to have generally lower maximum CBD plasma concentrations compared to canines, implying species-specific factors which affect bioavailability (10). Fasting versus fed states, cannabinoid ratios (CBD:THC), and dose also appeared to alter CBD pharmacokinetics (9). There are limited published studies evaluating CBD pharmacokinetics in cats (10–14), and even fewer using formulations containing known quantities of both CBD and THC (13, 14). With many cannabinoid products available to animal owners, each with unique chemical makeup, understanding potential interactions between cannabinoids and the resulting impact on plasma concentrations is essential.

The objective of this study is to determine the cannabinoid-plasma concentrations in cats for two different doses of a 20:1 CBD:THC cannabis herbal extract (CHE) previously evaluated in dogs (15). This study aims to develop a rational dosing regimen suitable for use in clinical trials evaluating the safety and efficacy of Cannabis herbal extracts (CHE) in cats.

## Materials and methods

### Cats

This study was approved by the Usask Animal Research Ethics Board (Animal Use Protocol 20,210,019). Twelve Domestic Shorthair (DSH) cats (four castrated males and eight spayed females) housed at the WCVM Animal Care Unit were used in this study. Ages ranged from 0.75–9 years and weighed 3.34–6.91 kg at the start of study. Health was assessed via history, physical examination, and complete blood count and chemistry profiles prior to study initiation, all cats were considered in good health. The standard diet was a nutritionally balanced commercial cat food offered twice daily in individual cat feeders, however food was withheld from cats for 12 h prior to dosing and offered again 2 h post-dose.

### Test item

CBD-enriched *Cannabis* herbal extract (CHE) with nominal concentrations of 20 mg CBD and 1 mg THC per mL in olive oil base (CanniMed) was provided from a licensed cannabis producer (Aurora Cannabis Inc.). All necessary regulatory approvals for experimental use of this CHE in cats was granted by Health Canada (Experimental Studies Certificate and Cannabis research exemption) prior to study initiation. A certificate of analysis was submitted by Aurora Cannabis Inc. for the batch of CanniMed used in the study.

### Pharmacokinetic (PK) study design

Cats were stratified by weight and sex, then randomly assigned to low (2 mg CBD + 0.1 mg THC/kg) or high (5 mg CBD + 0.25 mg THC/kg) dose groups (*n* = 6 per dose group). All cats were fasted for 12 h prior to the planned dosing time. On the dosing day, indwelling cephalic vein catheters were placed and 1–1.5 mL whole blood collected as a Time 0 sample. CHE dose volumes were based on Day −1 body weights and ranged from 0.33–0.69 mL (low dose) and 0.89–1.58 mL. Oral dose administration was performed using 1 mL or 3 mL syringes placed on the back of the cat’s tongue, followed by holding the cat’s mouth closed for 10–20 s or until swallowing was noted. Starting 2 h after dosing, and throughout the rest of the blood collection period, cats had free access to their normal diets via automated microchip feeders. During the intensive blood collection phase (first 8 h after dosing), treated cats were confined to a single room with free access to food and water, in order to facilitate regular blood collection and supervision. Cats were monitored post-dose for any adverse events (AEs) such as head shaking, hypersalivation, or vomiting.

Whole blood samples (2.0–2.5 mL) were taken via the catheters at the following nominal times; 0.5, 1, 1.5, 2, 3, 4, 6, 8, 12, 24, 32, and 48 h. The catheters were flushed with saline regularly to prevent clots from lodging. When taking samples, the first 0.5 mL of blood was discarded to prevent sample dilution from the saline flush. If the catheters became dislodged, kinked, or plugged, the blood sample was collected by jugular or alternate limb cephalic venipuncture. All blood samples were collected in labeled lithium heparin tubes and immediately refrigerated. Actual collection times were recorded for each sample. Whole blood samples were centrifuged at 1200 x G for 10 min. Plasma was separated via pipette into 200 μL aliquots and stored in Eppendorf Protein Lo-Bind microcentrifuge tubes and frozen at −80°C for up to 12 months prior to analysis.

### Adverse event and neurological assessment

Throughout the first 12 h of the pharmacokinetic study phase, cats were directly monitored for signs of vomiting, salivation, diarrhea, changes in mentation, hyperesthesia, or any other physical or neurological abnormality.

### Plasma sample preparation and LC–MS/MS analysis

The analytical method used for cannabinoid analysis in feline plasma was a version of an assay previously validated for canine plasma (15). Assay limit of detection (LOD) was 0.98 ng/mL for all analytes measured (CBD, THC, 6-OH-CBD, 7-OH-CBD, 11-OH-THC, and THC-COOH). Lower limit of quantification (LLOQ) was 1.97 ng/mL for all analytes except 7-OH-CBD (3.91 ng/mL).

### Pharmacokinetic analysis

Plasma concentration versus time data was analyzed for each cat using non-compartmental modeling (Phoenix WinNonLin, Certara, Princeton, NJ, United States). Final PK parameters were expressed as mean ± SD. Maximum plasma concentration (*C*_max_) and time to maximum concentrations (*T*_max_) were assessed by visual inspection of the C-T curves. Determination of the log-linear terminal rate constant, λ_z_, was based on the terminal slope (typically the last 4 quantifiable plasma samples, from 12–48 h post-dose) of the natural logarithmic C-T curve using linear regression analysis. However, for some cats with prolonged quantifiable plasma concentrations, an intermediate (β-phase) rate constant was determined from samples between *T*_max_ and 12 h. The β-phase half-life (T_1/2, β_) and terminal elimination (λ_z_) half-life (T_1/2, λz_) were calculated as ln2/β and ln2/λ_z_, respectively. The area under the C-T curve from 0 h to the last quantifiable plasma concentration (AUC_0-last_) was determined using the linear trapezoidal rule. In order to compare PK parameters between the low and high dose groups, C_max_ and AUC_0-last_ were dose-normalized (divided by dose administered). Apparent volume of distribution (V_d_/F) and apparent clearance (Cl_S_/F) were also derived.

### Statistical analysis

Dose-normalized *C*_max_ and AUC_0-last_ values for CBD and THC were compared between low and high dose groups with a two-sample *T*-test (Graphpad Prism 9.3, GraphPad Software, La Jolla, CA). Dose-normalized *C*_max_ and AUC_0-last_ were also compared between THC and CBD using a two-sample *T*-test. A *p* value of <0.05 was defined as the cutoff for statistical significance. Due to limited numbers of observations and variation in assessment procedures between dose groups, statistical evaluation of the neurological evaluations and adverse events was considered inappropriate; only incidence of findings is reported.

## Results

### CHE formulation

A single batch of CHE (CanniMed) was used for the entirety of this study and contained 19.5 mg CBD and 1.0 mg THC per mL (nominal concentrations of 20 and 1.0 mg/mL, respectively). Elemental impurities, mycotoxins, or pesticides were either not detected or not quantifiable.

### Dose administration and adverse events

**Figure 1:**
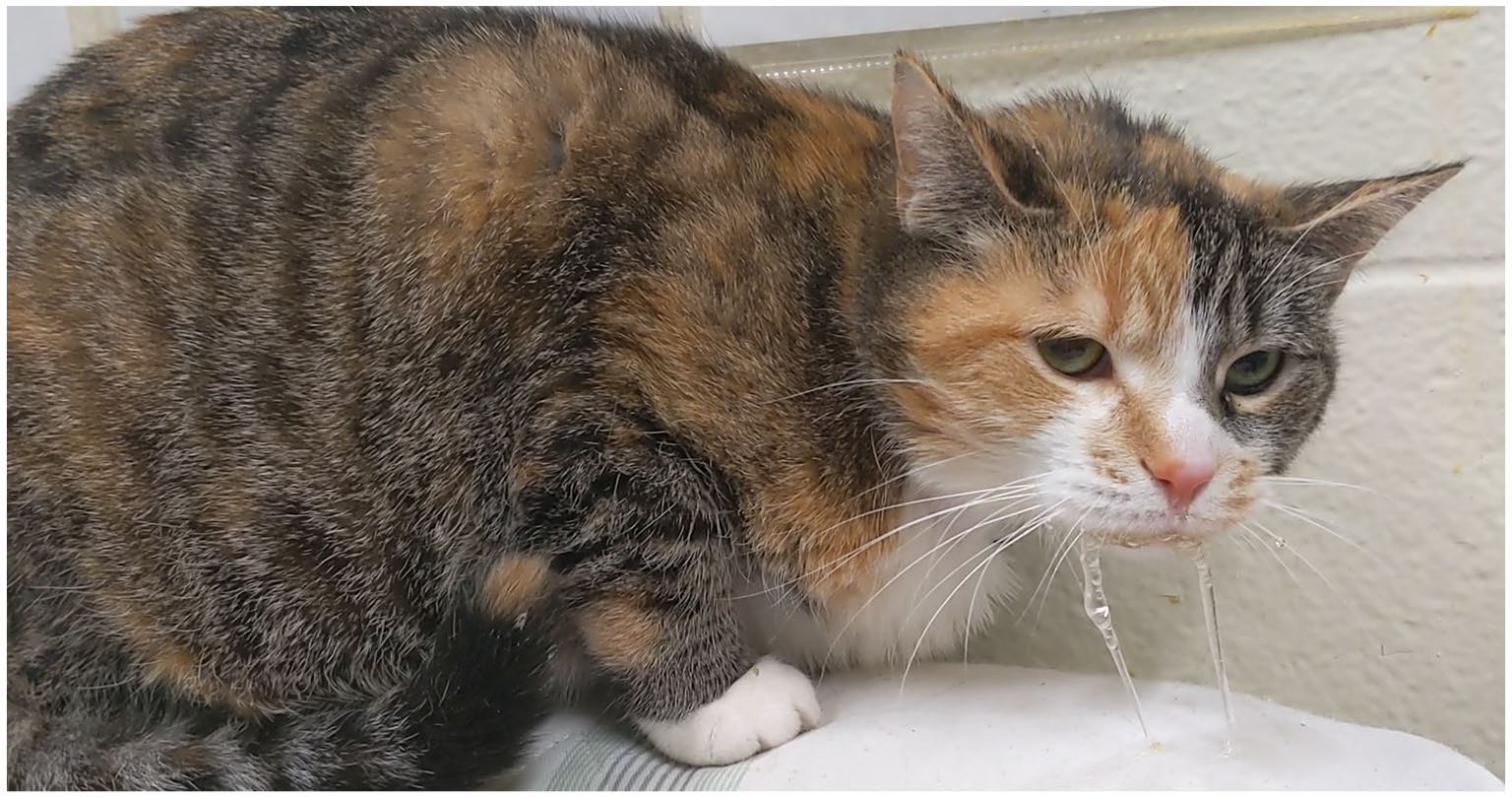
Cat demonstrating hypersalivation immediately following administration of a single 5 mg CBD/kg bw oral dose of 1:20 THC:CBD CHE formulation.

Administration of the CHE was generally well tolerated in the cats. To ensure swallowing of the CHE following dose administration, the cat’s mouth was held closed for 10–20 s or until swallowing was visualized to ensure swallowing. However, two cats in the high dose group had moderate hypersalivation within 2–10 min of dosing (see Figure 1). Following analysis of plasma samples by LC–MS/MS, it was noted that these two cats had substantially lower cannabinoid concentrations than the other cats in the high dose group. It is presumed that these cats swallowed only a fraction of the administered dose and may have expelled the remainder in the saliva or while licking their lips during salivation. However, because there was no way to verify or quantify that these cats did not receive the entire dose, their plasma concentrations were included in the data analysis. Cats were intensively monitored for the first 8 h after dosing; no cat vomited, regurgitated, or coughed up any CHE during this time. Due to demonstration of hyperesthesia in dogs after use of the same CHE doses and formulation (15), cats were carefully evaluated for any potential neurological or behavioural changes. No neurological abnormalities were observed and the cats did not exhibit sedation or altered mentation.

### Pharmacokinetic results

**Figure 2:**
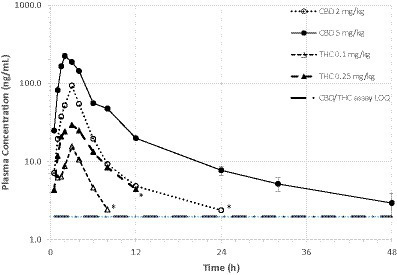
Mean plasma CBD and THC concentrations over time in fasted cats (*n* = 6/dosing group) receiving a single oral dose of 1:20 THC:CBD CHE formulation at 2 or 5 mg CBD/kg bw. Variance (s.d.) not shown due to overlapping error bars. ^*^Mean value based on only 2 quantifiable concentrations.

Mean ± SD plasma concentrations of CBD and THC by dose group are shown in Table 1. Variance within each dose group was very high, with CV% exceeding 100% at some time points. Mean plasma concentration versus time curves for CBD and THC in both dose groups are shown in Figure 2. In the high dose (5 mg CBD/kg bw) group, all 6 cats had plasma CBD concentrations above the limit of quantification (1.97 ng/mL) at all study time (including 48 h post-dose). For cats in the low dose (2 mg CBD/kg bw) dose group, CBD concentrations were only quantifiable up to 24 h post-dose. THC concentrations were only quantifiable (> 1.97 ng/mL) up to 4–12 h after dosing. The CBD metabolite 6-OH-CBD was quantifiable sporadically at various time points from 0.5–12 h post-dose, with the highest single concentration observed of 16.8 ng/mL. Other cannabinoid metabolites included in the assay (7-OH-CBD, 11-OH-THC, and THC-COOH) were not detectable in any plasma samples.

**Table 1:** Mean ± S.D. concentrations of CBD and THC in plasma for fasted Domestic Shorthair cats administered a single dose of CHE (*n* = 6 per dose group).

Time (h)	Low dose (2 mg CBD + 0.1 mg THC/kg bw)			High dose (5 mg CBD + 0.25 mg THC/kg bw)		
	CBD (ng/mL)	THC (ng/mL)	THC:CBD ratio	CBD (ng/mL)	THC (ng/mL)	THC:CBD ratio
0.5	7.1 ± 6.4 (5)	ND	–	24.9 ± 28.4	4.3 ± 3.0	0.17
1.0	19.6 ± 21.9	6.3 ± 1.3 (2)	–	81.9 75.3	11.8 ± 9.2 (4)	0.14
1.5	37.6 ± 27.9	6.4 ± 4.1	0.32	165.1 ± 135.2	20.7 ± 15.7	0.13
2.0	52.1 ± 24.9	8.8 ± 4.1	0.17	223.8 ± 188.1	24.3 ± 22.8	0.11
3.0	93.6 ± 90.4	15.6 ± 13.2	0.17	187.4 ± 157.4	29.4 ± 20.4	0.16
4.0	54.6 ± 46.3	10.7 ± 9.2	0.20	143.8 ± 113.3	24.9 15.3	0.17
6.0	19.6 ± 11.6	4.7 2.4	0.24	55.8 ± 47.6	13.3 ± 10.3	0.24
8.0	9.3 ± 3.1	2.5 ± 0.5 (2)	0.26	47.6 ± 55.5	8.4 ± 6.9 (4)	0.18
12.0	4.9 ± 2.1	ND	–	19.9 ± 16.9	4.4 ± 1.3 (2)	–
24.0	2.4 ± 0.3 (2)	ND	–	7.7 ± 3.1	ND	–
32.0	BLOQ	ND	–	5.2 ± 2.2	ND	–
48.0	BLOQ or ND	ND	–	3.0 ± 0.7 (6)	ND	–

Numbers in parentheses indicate number of quantifiable plasma concentrations at the time point. BLOQ, below limit of quantification (LLOQ = 1.97 ng/mL), ND, not detectable (LOD = 0.98 ng/mL), NA, not available.

CBD and THC pharmacokinetic (PK) parameters are shown in Table 2. PK parameters were not derived for 6-OH-CBD due to the limited number of quantifiable concentrations observed. Dose-adjusted *C*_max_ and AUC_last_ (i.e., *C*_max_ or AUC_0-last_/dose) values were not statistically significantly different between the low and high dose groups for either CBD or THC. However, when the dose-adjusted parameter (*C*_max_ or AUC_last_) results were combined for both dose groups and compared between cannabinoids, the THC dose-adjusted parameter was statistically significantly higher than the CBD dose-adjusted parameter (*p* < 0.01). Apparent volume of distribution (V_d_/F) and apparent clearance (Cl_S_/F) were calculated but not reported due to the unknown bioavailability (*F*) and high variance observed.

**Table 2:** Mean (SD) cannabinoid PK parameters in fasted Domestic Shorthair cats (n = 6/dose group) receiving a single oral dose of 1:20 THC:CBD CHE at 2 or 5 mg CBD/kg bw.

Cannabinoid	Dose (mg/kg)	*T*_max_ (h)	*C*_max_ (ng/mL)	Dose-Adj. *C*_max_ (ng/mL per mg/kg dose)^a^	T_1/2 β_ (h)^b^	T_1/2 λ_ (h)^c^	AUC_0-last_ (ng*h/mL)	Dose-Adj. AUC_0-last_ (ng*h/mL per mg/kg)^a^
CBD	2	2.4 (0.7)	111.2 (79.0)	55.6 (39.5)	2.9 (2.0)	7.7 (1.0)	344 (183)	170.8 (91.4)
	5	2.5 (0.8)	214.1 (182.8)	42.8 (36.6)	2.5 (0.4)	17.1 (5.8)	1,293 (970)	258.6 (194.0)
Combined CBD dose groups				49.2^A^ (36.9)	Combined CBD dose groups			214.7^A^ (151.7)
THC	0.1	2.9 (1.6)	17.1 (12.0)	170.6 (120.3)	2.4 (1.5)	NA	52.5 (32.0)	525.4 (320.3)
	0.25	2.7 (0.8)	27.9 (21.9)	111.8 (87.8)	2.0 (0.6)	NA	147 (117)	587.3 (465.8)
Combined THC dose groups				141.2^B^ (105.0)	Combined THC dose groups			556.3^B^ (378.3)

a Dose-adjusted value (parameter value divided by mg/kg dose).

b T1/2 β phase, from Tmax – 12 h post-dose.

c T1/2 λ (terminal elimination) phase, from 12–48 h post-dose.

*T*_max_, time to maximum concentration, *C*_max_, maximum concentration, AUC, area under the plasma concentration versus time curve, T1/2, half-life. CBD and THC statistical analysis: Differing alphabetical superscripts in each column indicate statistically significant (*p* < 0.05) differences between mean values (two-sample *T*-test). NA, not applicable.

## Discussion

There is limited and sometimes conflicting information regarding pharmacokinetics and pharmacodynamics of cannabinoids in feline medicine, which makes recommending specific dose regimens challenging for veterinarians. Delta-9-tetrahydrocannabinol (THC) and cannabidiol (CBD) are two of the 120 cannabinoids discovered from the Cannabis plants that have been studied most extensively, particularly in recent years. This study investigated the pharmacokinetics of CBD and THC after administration of a single dose of 1:20 THC:CBD cannabis herbal extract in fasting cats, with plasma concentrations collected over a 48-h period. The CHE doses selected for this study (2 or 5 mg CBD; 0.1 or 0.25 mg THC/kg bw) were based on those used for the same formulation in dogs which resulted in minimal adverse effects (15).

The PK parameters derived in this study was broadly similar to others evaluating cannabinoid PK in cats. Oral CBD administration in fasting cats has consistently demonstrated rapid absorption, with published mean *T*_max_ values typically reported around 2 h (10–13). Mean CBD *T*_max_ values in this study were 2.4 and 2.5 h for the 2 and 5 mg CBD/kg bw doses, respectively. Previously published studies in cats noted rapid CBD elimination half-life values of 1.5–4 h (10, 11, 13), but the elimination half-lives were typically much longer in this study (up to a mean of 17.1 h in the 5 mg/kg dose group). This difference may be attributable to the dose regimen and plasma sampling schedule. Another study (12) using higher CBD doses (e.g., 5 mg/kg and up) resulting in quantifiable plasma concentrations up to 48 h, noted a similarly prolonged terminal elimination phase (λ_z_) and thus longer elimination half-lives. Similar differences in CBD elimination half-lives have been noted in canine studies using sampling schedules beyond 24 h (15). Despite the relatively long terminal elimination half-life for CBD in the 5 mg/kg dose group, the likelihood of clinically-relevant bioaccumulation occurring with repeated daily dosing appears to be low. The rapid T_1/2β_ (from *T*_max_ to 24 h) for both CBD and THC (values comparable to the elimination half-life values reported in other animal studies), leaves very low cannabinoid concentrations when the terminal elimination phase begins.

Peak CBD plasma concentrations (*C*_max_) and overall exposure (AUC) in cats appear to vary considerably between studies. Wang et al. (13) used a similar dose (1.37 mg CBD/kg bw) and cannabinoid ratio (1:27 THC:CBD) as those used in this study, yet the mean *C*_max_ in that study was substantially higher than after administration of 2 mg CBD/kg in this study (282.0 ± 149.4 ng/mL compared with 111.2 ± 79.0 ng/mL, respectively). The cats in both studies were fasted; however, the vehicle for cannabinoid delivery was different (food-based paste versus olive oil-based cannabis herbal extract). Another study administering a pure CBD formulation (11) to fasting cats at dose of 5 mg/kg bw reported comparable mean *C*_max_ and AUC values (269 ± 334 ng/mL; 921 ± 1,003 ng*h/mL) cats administered the same dose in this study (214.2 ± 182.8 ng/mL; 1,293 ± 970 ng*h/mL). Finally, other recent studies (10, 12) using comparable CBD doses reported mean *C*_max_ and AUC values that were approximately 20–50% of the values determined in this study.

Cannabinoids are lipophilic and are considered to have poor oral bioavailability, but display increased absorption in fed rather than fasting states in humans (16, 17). A recent crossover study (11) in cats demonstrated similar results with CBD exposure in the fed state being statistically significantly higher than in the fasting state. Other factors impacting oral bioavailability may include the presence of additional cannabinoids (such as THC) in the formulation. While the cannabinoid combination leading to a pharmacodynamic synergism (so-called “entourage effect”) has been postulated in humans (4, 18) and animals (19, 20), the potential for THC to modulate CBD pharmacokinetic properties (such as bioavailability or clearance) cannot be ruled out. For example, another study in cats (14) using cannabis formulations with varying ratios of CBD and THC demonstrated significantly higher plasma CBD concentrations when combined with THC at a 1.5:1 CBD:THC ratio, compared to a 25:1 ratio.

As expected, the plasma concentrations and exposure of CBD and THC were elevated in the high dose group (5 mg CBD/kg bw) compared to the lower dose (2 mg CBD/kg bw). The increase in *C*_max_ and AUC_0-last_ for both CBD and THC was roughly proportional to the increase in dose (2.5 fold higher). After standardizing by the dose administered (*C*_max_/dose and AUC_0-last_/dose), there were no statistically significant differences between the two dose groups for either parameter, either for CBD or THC. This suggests linear kinetics over the dose range utilized in this study, and is consistent with results from another recent cannabinoid PK study in cats utilizing a larger dose range (12). However, it was readily apparent that although the formulation used was a 1:20 THC:CBD extract (i.e., the THC dose comprised only 5% of the CBD dose), THC plasma concentrations were consistently higher than 5% of the CBD concentrations (Table 1). When dose-adjusted PK parameters from both dose groups are combined, the *C*_max_ and AUC_last_ for THC were statistically significantly higher than the CBD parameters (Table 2). Increased dose-adjusted THC plasma concentrations (relative to dose-adjusted CBD concentrations) were also demonstrated in other cannabinoid studies in cats using varying ratios of THC and CBD (13, 14), and in our previous canine trial (15) using the identical formulation as this study. The reason for the (relatively) elevated THC concentrations compared with CBD is not immediately obvious. THC may have increased relative bioavailability, or decreased systemic clearance, compared to CBD in cats. For example, a cannabinoid study in rats hypothesized that CBD inclusion may lead to saturation of cytochrome P450 enzymes or transmembrane proteins, thus reducing the metabolism or transport of THC (20). Alternatively, it may be that at the 20-fold difference between THC (0.1 and 0.25 mg/kg) and CBD (2 and 5 mg/kg) doses used in this study, the kinetics are not linear. If so, comparisons of “dose-adjusted” PK parameters between THC and CBD would not be valid. Further studies would be necessary to assess if this suspected THC “overperformance” (relative to CBD) in cats is consistent across varying CBD:THC ratios and doses.

Although multiple CBD and THC metabolites were included in the analytical method, only the CBD metabolite 6-OH-CBD was quantifiable at any sampling times. The other metabolites (7-OH-CBD, 11-OH-THC, and COOH-THC) were not detected in any samples. Another feline cannabinoid PK study did detect low concentrations of 11-OH-THC in feline plasma (14), but had administered significantly higher THC doses than in this study. Analytical methods used in most other previously published feline cannabinoid PK studies did not include cannabinoid metabolites. However, based on results from this study it is unlikely that such metabolites would have been detected.

The CBD and THC plasma concentrations from this feline study were generally lower than those observed in a previous canine study (15) using the same 1:20 THC:CBD formulation (CanniMed) and doses (2 and 5 mg CBD/kg bw). Lower cannabinoid concentrations could be due to species-specific pharmacokinetics in cats, such as inherently decreased absorption or increased rate of clearance compared to dogs. An alternative explanation is that technical challenges associated with oral administration oil-based extracts in cats may also be a factor in the reduced plasma concentrations. Quite simply, it is generally more difficult to administer oral substances to cats than to dogs. Study investigators ensured that the entire dose was administered into the cat’s oral cavity, and waited for visual confirmation of swallowing before releasing the cats mouth. However, cats are notorious for “spitting up” oral medications which they conceal in their oral cavity, and it could not be confirmed that all cats swallowed the entire CHE dose. While no cat regurgitated or vomited after dosing, two cats (both in the high dose group) experienced excessive salivation within a couple minutes of dosing and had substantially lower cannabinoid concentrations than the other four cats in this dose group. Any oil-based CHE retained in the oral cavity may have prompted the cat to salivate, and subsequently been expelled from the mouth. However, while these two cats clearly hypersalivated, loss of cannabinoids in the saliva cannot be confirmed and therefore the results from these cats were not excluded from the analysis.

Challenges with oral dosing of felines may also contribute to the high degree of variance (S.D.) in PK parameters in each dose groups. CBD and THC plasma concentrations varied dramatically between individual cats in the same dose group, a finding observed in similar feline cannabinoid PK studies (5, 11). Alternatively cats may simply have inherently high inter-individual variability (or intra-individual variability after multiple doses) in cannabinoid kinetics. Such variance makes developing a therapeutic dosing regimen difficult. Cannabinoid therapeutic drug monitoring is typically not available for veterinary patients, and thus the veterinarian must dose empirically and adjust based on clinical response.

In summary, fasting cats administered a single oral dose of a 1:20 THC:CBD oral extract at 2 or 5 mg CBD/kg demonstrated no significant adverse effects and plasma concentrations generally comparable to other published studies in cats. CBD and THC concentrations increased in a linear fashion over the dose range, but THC concentrations were significantly higher than CBD concentrations when adjusted for dose administered. Plasma concentrations were highly variable between individual cats in the same dose group. While the dose regimens used in this study appear suitable for use in future feline clinical studies, veterinarians should not expect uniform responses when administering the same CHE dose to different cats.

## Data availability statement

The datasets presented in this article are not readily available because proprietary information relating to cannabis formulations used cannot be disclosed. Requests to access the datasets should be directed to AC, al.chicoine@usask.ca.

## Ethics statement

The animal study was approved by University of Saskatchewan Animal Research Ethics Board, adhering to the Canadian Council on Animal Care guidelines for humane animal use (Animal Use Protocol Number 20210019). The study was conducted in accordance with the local legislation and institutional requirements.

## Author contributions

CL: Data curation, Formal analysis, Writing – original draft, Writing – review & editing. KM: Data curation, Investigation, Writing – original draft. MM-P: Investigation, Writing – original draft. SV: Formal analysis, Methodology, Writing – review & editing. JA: Conceptualization, Methodology, Writing – review & editing. AC: Conceptualization, Data curation, Formal analysis, Funding acquisition, Investigation, Methodology, Project administration, Supervision, Writing – original draft, Writing – review & editing.
